# Internalization, distribution, and activity of peptide H2 against the intracellular multidrug-resistant bovine mastitis-causing bacterium *Staphylococcus aureus*

**DOI:** 10.1038/s41598-019-44459-x

**Published:** 2019-05-28

**Authors:** Xiao Wang, Da Teng, Xiumin Wang, Ya Hao, Huixian Chen, Ruoyu Mao, Jianhua Wang

**Affiliations:** 1 Key Laboratory of Feed Biotechnology, Ministry of Agriculture, Beijing, 100081 People’s Republic of China; 2 Gene Engineering Laboratory, Feed Research Institute, Chinese Academy of Agricultural Sciences, Beijing, 100081 People’s Republic of China

**Keywords:** Antimicrobials, Cellular imaging

## Abstract

Bovine mastitis is mainly caused by *Staphylococcus aureus*, which is difficult to eliminate, prone to escape from antibacterial agents, and may cause recurring infections due to the intracellular nature of its infection and multidrug resistance. In this study, the intracellular activities of the NZ2114 derivative peptide H18R (H2) against methicillin-resistant *S. aureus* (MRSA) and multidrug-resistant bovine *S. aureus* strains were investigated in bovine mammary epithelial MAC-T cells and mouse mammary glands. The minimum inhibitory concentrations of H2 against *S. aureus* were 0.5‒1 μg/ml; H2 displayed a lower cytotoxicity than its parental peptide NZ2114 (survival rates of MAC-T cells: 100% [H2 treatment] vs 60.7% [NZ2114 (256 μg/ml) treatment]). H2 was internalized into MAC-T cells mainly via clathrin-mediated endocytosis, and distributed in the cytoplasm. The intracellular inhibition rates against MRSA ATCC43300, the mastitis isolates *S. aureus* CVCC 3051 and E48 were above 99%, 99%, and 94%, respectively; these were higher than those in case of vancomycin (23–47%). In the mouse model of *S. aureus* E48-induced mastitis, after treatment with 100 μg of H2 and vancomycin, bacterial numbers in each mammary gland were reduced by 3.96- and 1.59-log CFU, respectively. Additionally, similar to NZ2114 and vancomycin, H2 alleviated the histopathological damage of the mammary tissue and polymorphonuclear neutrophil infiltration in the alveoli. These results suggest that H2 can be used as a safe and effective candidate for treating *S. aureus*-induced mastitis.

## Introduction

As an opportunistic pathogen with a broad range of hosts, *Staphylococcus aureus* can lead to the acute and chronic infections in humans or farm animals worldwide^1^; bovine mastitis is one of the most serious concerns with regards to milk production^2^. In China, the average incidence rate of bovine mastitis is about 33%; it leads to annual economic losses of ¥15–45 billion, which seriously affects the development of the dairy industry^3–5^. Meanwhile, accumulating evidences have demonstrated that *S. aureus* can internalize into host cells, such as mammary gland epithelial cells, and form an intracellular pathogen pool, which destroys the epithelial barrier and causes further damages, finally leading to the recurrence of bovine mastitis^6,7^.

Although antibiotic therapy is a common method to eliminate intracellular *S. aureus* reservoirs in bovine mastitis-infected tissues, they have a low treatment efficiency and are associated with a high infection recurrence rate due to their low permeation ability in mammary glands^7–9^. Additionally, the overuse of antibiotics in developing countries can trigger a series of serious problems, such as the destruction of polymorphonuclear leukocytes and immune function in mammary glands, as well as enhanced bacterial resistance^10^. In a recent investigation in China, among 219 *S. aureus* strains those were isolated from bovine mastitis cases from six provinces (Beijing, Shandong, Shanxi, Inner Mongolia, Xinjiang, and Zhejiang), more than 70% showed resistance to various antibiotics^11^. Moreover, *S. aureus* strains isolated from mastitis-infected samples in other countries such as North and South America, Asia, and Malta, have been reported to be resistant to different antibiotics^12^. Therefore, there is an urgent need to find effective antibiotic alternatives for the treatment of bovine mastitis.

Antimicrobial peptides (AMPs) are promising antibiotic alternative candidates, owing to their multiple modes of action such as membrane permeabilization, binding to DNA, and inhibition of protein synthesis^13,14^. The first fungal defensin-plectasin, isolated from *Pseudoplectania nigrella*, displays a potent activity against Gram-positive bacteria including *S. aureus* by binding to the cell wall precursor-lipid II^15,16^. Both NZ2114 (D9N, M13L, and Q14R) and MP1102 (D9Q, M13V, and Q14R), which are derivatives of plectasin, have a stronger activity than their parent; this may be due to their more positive net charges and higher hydrophobicity^17–20^. Additionally, similar to daptomycin and vancomycin, NZ2114 and MP1102 display a potent intracellular activity against *S. aureus* in THP-1 monocytes^21^, RAW 264.7 macrophages^22^, and mammary epithelial cells^23^, and can reduce the *S. aureus* load in the mammary glands of mice^23^. However, NZ2114 and MP1102 are more toxic towards mouse macrophages than vancomycin due to their high hydrophobicity^22^. Therefore, in our previous study, H2 (H18R), a novel NZ2114-derived peptide, was designed by replacing the histidine at position 16 with arginine to reduce its cytotoxicity; the results showed that H2 almost retained its original antibacterial activity against *S. aureus* and showed high stability in different environments^24^. Therefore, it is speculated that H2 may serve as a potential antibacterial agent against infections caused by *S. aureus*.

In this study, based on the bactericidal activity of H2 against planktonic *S. aureus*, the toxicity, translocation, subcellular distribution, and quantification of H2 in the bovine mammary epithelial cells (MAC-T) were investigated. Additionally, the intracellular activity of H2 was evaluated against one multidrug-resistant *S. aureus* strain methicillin-resistant *S. aureus* (MRSA) ATCC43300, and two clinical mastitis isolates: *S. aureus* CVCC3051 and E48) (Supplementary Tables S1 and S2) in MAC-T cells; the therapeutic efficacy of H2 was also investigated in an *S. aureus*-infected mouse mastitis model.

## Results

### Intracellular invasion abilities of three different *S. aureus* strains

The antimicrobial susceptibility and typing results showed that the bovine mastitis isolates of *S. aureus* CVCC3051 and E48 can resist multiple antibiotics such as bacitracin, sulfisoxazole, streptomycin, or ampicillin, and they are assigned to *spa* type t3297 and t3583 (Supplementary Tables S1 and S2).

**Figure 1:**
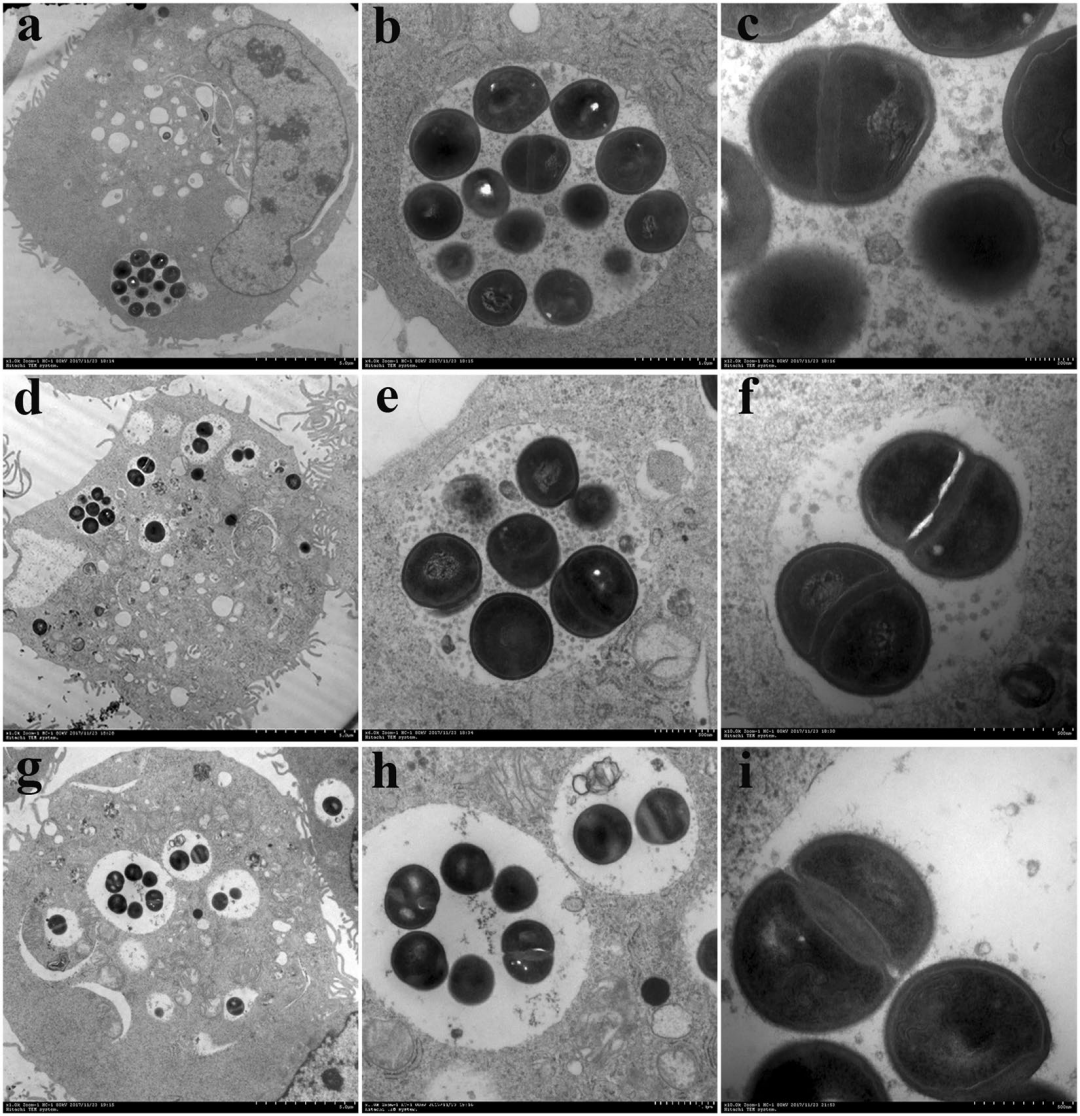
Morphology of *S. aureus* cells within MAC-T cells. The MAC-T cells were challenged with MRSA ATCC43300 (**a**–**c**), clinical isolate of *S. aureus* CVCC3051 (**d**–**f**), and clinical isolate of *S. aureus* E48 (**g**–**i**) at an MOI of 100:1 (bacteria to macrophages), and observed by TEM after 0.5 h of invasion. All pathogens entered the MAC-T cells and were localized in the vacuoles (**a**) and the cytoplasm (**a**,**d**,**g**,**h**). The bacteria could proliferate in the host cells (**c**,**f**,**i**).

To determine the intracellular activities of the peptides against *S. aureus*, MAC-T cells were infected with MRSA ATCC43300 and bovine mastitis isolates (*S. aureus* CVCC3051 and E48); the results showed that the pathogens entered the MAC-T cells and were localized in the vacuoles (Fig. 1a) and the cytoplasm, respectively (Fig. 1a,d,g,h). Additionally, although some bacteria could be killed in the vacuoles (Fig. 1b,c,e), the dividing intact *S. aureus* cells were also observed in the host cells (Fig. 1c,f,i), indicating that the three *S. aureus* strains can survive and proliferate in MAC-T cells.

### Cathepsin B had no effect on the extracellular activities of H2

The influence of the lysosomal proteinase cathepsin B on the activity of H2 was tested by determining the minimum inhibitory concentrations (MICs). After treatment with cathepsin B, the MICs of H2 and NZ2114 against MRSA ATCC43300 (0.25–1 μg/ml) and the *S. aureus* strains CVCC3051 and E48 (0.5–1 μg/ml) did not change (Supplementary Table S3), indicating that cathepsin B has no influence on the antibacterial activity of H2.

### H2 displayed a lower cytotoxicity

**Figure 2:**
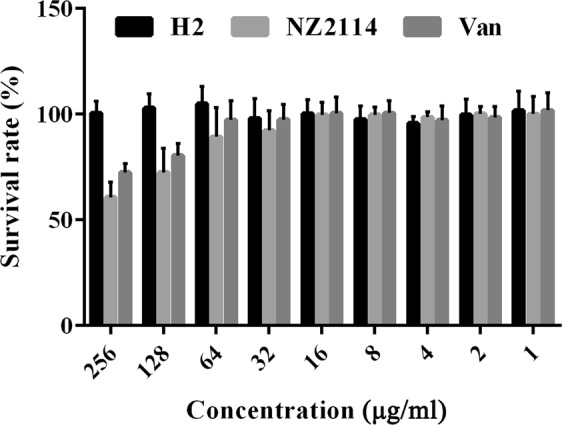
Viability of MAC-T cells incubated with NZ2114 and H2 for 24 h. MAC-T cells were incubated with 1–256 μg/ml for 24 h, and subsequently, cell viability was determined using the 3-(4,5-dimethylthiazol-2-yl)-2,5-diphenyltetrazolium bromide (MTT) assay. Results are represented as the means ± standard deviations (SDs) for three experiments. After exposure to 256 μg/ml H2, NZ2114 and vancomycin, the survival rates of MAC-T cells were 100%, 60.7% and 72.4%, respectively.

The viability of MAC-T cells was 100% when treated with 256 μg/ml H2, indicating that H2 shows no toxicity towards MAC-T cells. However, after exposure to 256 μg/ml NZ2114 and vancomycin, the survival rates of MAC-T cells were only 60.7% and 72.4%, respectively (Fig. 2), which was consistent with the findings of our previous study^21^. These results demonstrated that H2 is a safer candidate drug than vancomycin and NZ2114.

The effect of H2 on the cell membrane integrity was determined by the fluorescent dye propidium iodide (PI), which is blocked at the outside of intact membranes, but can penetrate damaged cell membranes and intercalate with nucleic acids. Therefore, the fluorescent intensity of PI indicates the degree of cell membrane integrity^22^. The result showed that after treatment with 250 μg/ml H2 and NZ2114, the amounts of PI-stained cells were only 2.83% and 3.85%, respectively (Supplementary Fig. S1). This suggests that H2 did not notably damage the cell membranes of the MAC-T cells.

### H2 was internalized and distributed in the cytoplasm of MAC-T cells

**Figure 3:**
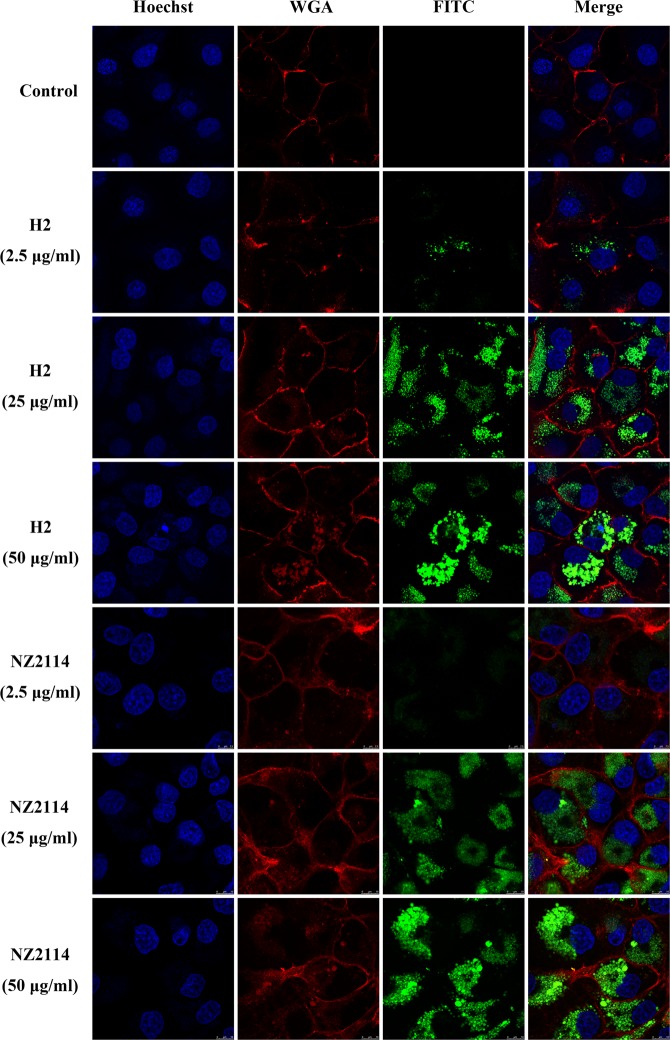
Cellular distribution of FITC-H2 and FITC-NZ2114 in MAC-T cells. The cells were incubated with 2.5, 25, and 50 μg/ml FITC-H2 and FITC-NZ2114 at 37 °C for 24 h, and then washed; they were then analyzed by confocal microscopy. Cell membranes and nuclei were stained with WGA-conjugated Alexa Fluor 555 (red) and Hoechst 33342 (blue), respectively. The FITC-peptides inside the cells display a green fluorescence. H2 and NZ2114 could enter the MAC-T cells in a dose-dependent manner and were distributed in the cytoplasm in a punctate manner.

The ability of H2 to enter host cells was evaluated by labeling the peptides and co-incubating them with MAC-T cells. The results showed that H2 and NZ2114 could enter the MAC-T cells in a dose-dependent manner and were distributed in the cytoplasm. At a low concentration of 2.5 μg/ml, fluorescein isothiocyanate (FITC)-H2 and FITC-NZ2114 could be very weakly observed in the cells (Fig. 3). With the increase in the concentration of the peptides (ranging from 25 to 50 μg/ml), the fluorescent intensity of FITC-H2 and FITC-NZ2114 was extremely enhanced in cells; the internalized peptides were accumulated in the cytoplasm. Moreover, the FITC-labeled peptides were distributed in the cytosol in a punctate manner, indicating that the peptides may be internalized by endocytosis.

**Figure 4:**
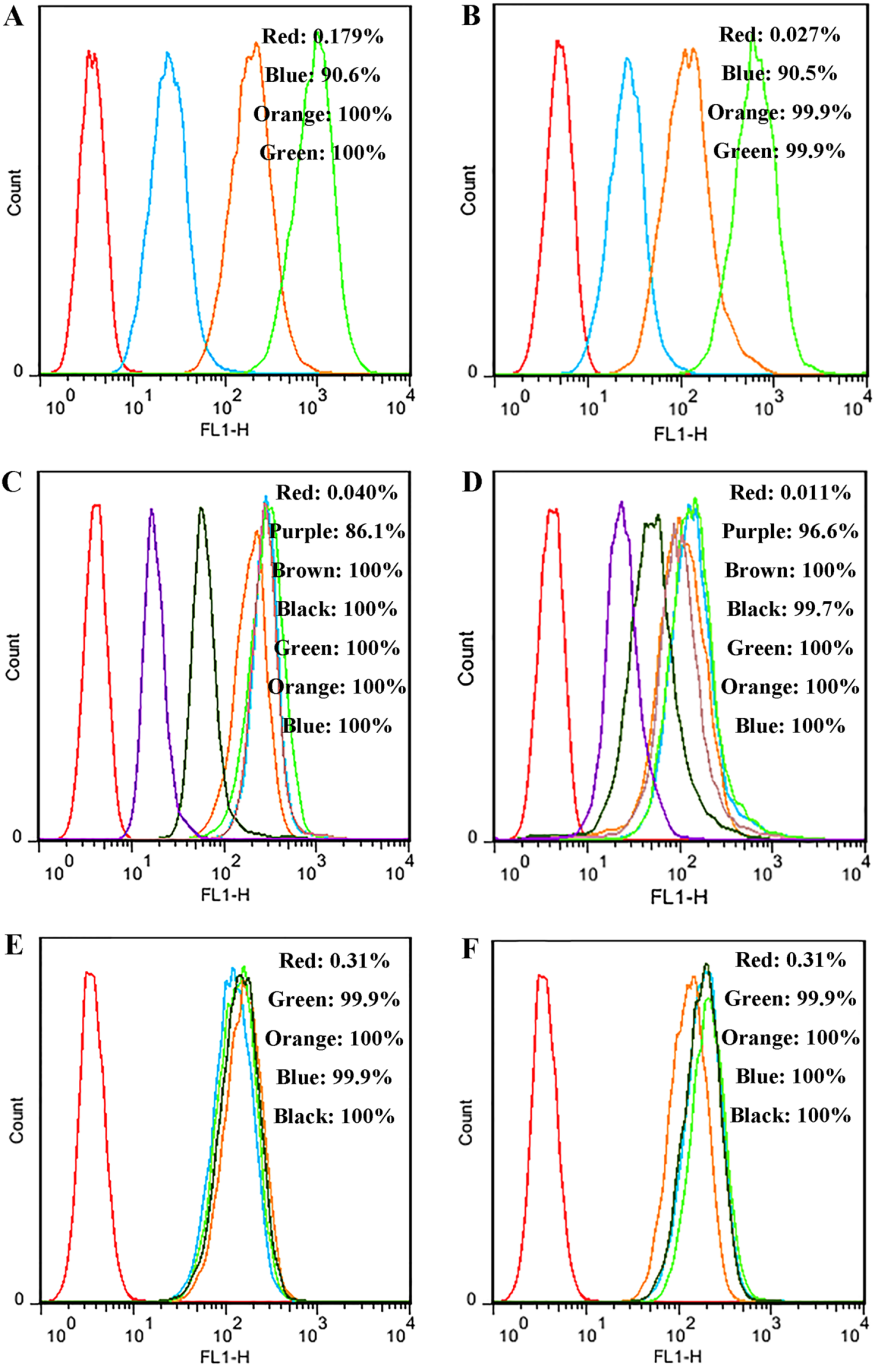
Quantification of H2 and NZ2114 in MAC-T cells and the preliminary mechanisms of their cellular uptake. (**A**,**B**) Analysis of FITC-labeled peptide uptake by flow cytometry. The cells were incubated for 24 h with FITC-H2 (**A**) and FITC-NZ2114 (**B**) at 37 °C prior to washing and quantification of peptide uptake. Red line: control; blue line: 0.25 μg/ml; orange line: 2.5 μg/ml; green line: 25 μg/ml. (**C**,**D**) Preliminary mechanism of the cellular uptake of FITC-labeled peptides. The cells were pretreated with different endocytosis inhibitors at 37 °C for 1 h prior to the addition of FITC-H2 (**C**) and FITC-NZ2114 (**D**). The uptake of peptides was quantified by flow cytometry. Red line: control; purple line: 4 °C; brown line: nocodazole; black line: chlorpromazine; green line: MβCD; orange line: amiloride; blue line: 25 μg/ml peptide. (**E**,**F**) Internalization of FITC-H2 (**E**) and FITC-NZ2114 (**F**) in MAC-T cells treated with soluble glycosaminoglycans (heparin, chondroitin sulfate, and dextran sulfate). MAC-T cells were incubated for 6 h with 25 μg/ml of the FITC-labeled peptides in the presence of 10 μg/ml of glycosaminoglycans. The cells were then washed and analyzed by flow cytometry. Red line: control; green line: heparin; blue line: chondroitin sulfate; orange line: dextran sulfate; black line: 25 μg/ml peptide.

To quantify the uptake efficiency of the peptides, the treated MAC-T cells were further analyzed by flow cytometry. After treatment with 0.25, 2.5, and 25 μg/ml FITC-H2, the percentages of cells showing fluorescence were 90.6%, 100%, and 100%, respectively (Fig. 4A), which is almost identical to those in case of NZ2114 (90.5–99.9%) (Fig. 4B). These data indicated that similar to NZ2114, H2 could enter the cells in a concentration-dependent manner and accumulate within MAC-T cells.

### Intracellular uptake of H2 occurred mainly by clathrin-mediated endocytosis

Low temperatures and different inhibitors of clathrin-mediated endocytosis, micropinocytosis, lipid rafts, and microtubule polymerization^25–27^ were used to investigate whether the peptides entered the MAC-T cells by endocytosis. The proportion of cells with fluorescence labeling of H2 and NZ2114 were reduced by 13.9% and 3.4%, respectively, and the fluorescence intensity of the cells decreased sharply (Fig. 4C,D), indicating that low temperature may have an inhibitory effect on peptide uptake^28^. In the inhibitor-treated groups, the fluorescence intensity of the cells treated with chlorpromazine (an inhibitor of clathrin-mediated endocytosis) decreased sharply; however, the fluorescence intensity did not change following treatment with amiloride (an inhibitor of macropinocytosis), methyl-β-cyclodextrin (MβCD) (a disruptor of lipid rafts), and nocodazole (an inhibitor of microtubule polymerization) (Fig. 4C,D).

### Internalization of H2 did not require the surface receptor

The effects of soluble glycosaminoglycans on the internalization of the peptides were tested to explore whether the uptake of the peptides depends on the surface receptors. As shown in Fig. 4E,F, the entrance of H2 and NZ2114 into MAC-T cells was not prevented by heparin, chondroitin sulfate, and dextran sulfate at a concentration of 50 μg/ml.

### H2 had potent intracellular bacteriostatic efficacy in MAC-T cells

**Figure 5:**
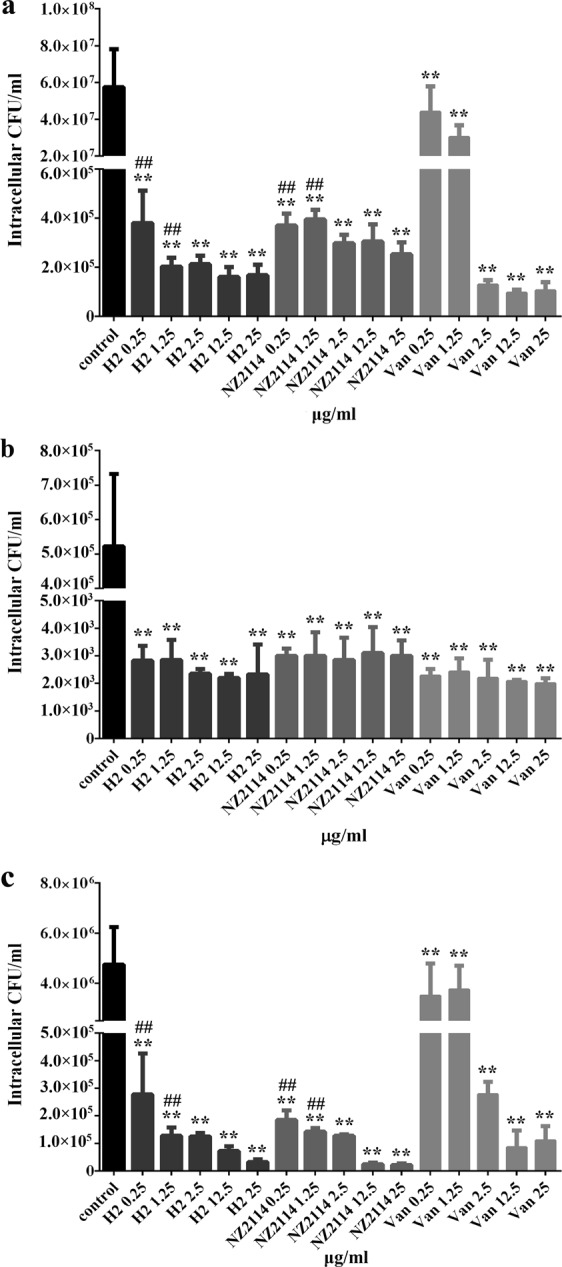
Intracellular activity of H2 and NZ2114 against *S. aureus* in MAC-T cells. Intracellular activities of H2, NZ2114, and vancomycin against MRSA ATCC43300 (**a**), and the clinical isolates *S. aureus* CVCC3051 (**b**) and E48 (**c**) were compared to those in case of the PBS-treated group. Van, vancomycin. Statistical analyses were performed using IBM SPSS Statistics 21.0. The analyses were measured by one-way ANOVA, with Duncan’s multiple comparisons test. A probability value of < 0.05 was considered significant. (*) indicates significance between the control and treatment groups. *P < 0.05; **P < 0.01. (#) indicates significance between the AMP- and Van-treated groups, with the same drug dose. ^#^*P* < 0.05; ^##^*P* < 0.01. The results are represented as the mean ± SD of three independent experiments performed in triplicate.

The intracellular activity of H2 (0.25–1.25 μg/ml) against *S. aureus* were assessed by means of a 24-hour dose-response study in MAC-T cells. As shown in Fig. 5, both H2 and NZ2114 effectively reduced the proportion of internalized *S. aureus* cells. In case of MRSA ATCC43300, the inhibition rates of intracellular bacteria were over 99% in the peptide-treated groups. The bacterial inhibition rates in the vancomycin-treated groups were 23% (at a concentration of 0.25 μg/ml) and 47% (at a concentration of 1.25 μg/ml), which were significantly lower than those in the peptide-treated groups (Fig. 5a). The killing efficiency of the peptides against *S. aureus* E48 was over 94%, which was slightly lower than that in case of MRSA ATCC43300. However, only 21–26% of intracellular *S. aureus* E48 cells were killed by vancomycin (Fig. 5c). Comparably, after 24 h of treatment with H2, NZ2114, and vancomycin, more than 99% of the intracellular *S. aureus* CVCC3051 cells were killed (Fig. 5b). Notably, the intracellular antibacterial activities of H2 and NZ2114 were higher than those of vancomycin at low concentrations. These results suggested that H2 and NZ2114 have more potent bacteriostatic effects than vancomycin against intracellular MRSA ATCC43300 and the clinical isolates CVCC3051 and E48; they elicited these effects in a dose-independent manner.

### H2 had a high therapeutic efficacy in mice

**Figure 6:**
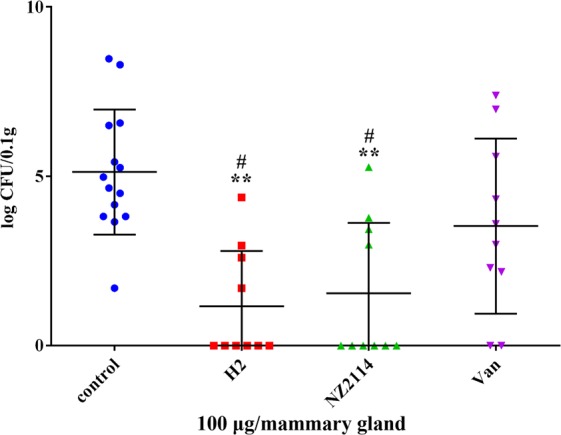
Activity of H2 and NZ2114 in the murine mastitis model. The mice were administered with intramammary injections with the clinical isolate *S. aureus* E48 (1 × 10^5^ CFU in 100 μl of PBS per mammary gland). Four hours later, the glands were injected with 100 μg of the peptides or vancomycin. Twenty-four hours after the treatment, bacterial loads in the mammary glands were counted. Each point represents data from a single mammary gland. Mean values are presented, n = 10 or 14. The error bars indicate the SD. Van, vancomycin. Zero in the *y*-axis for the peptides and vancomycin indicates that there are no bacterial colonies on the counting plate. Statistical analyses were performed using IBM SPSS Statistics 21.0. The analyses were evaluated by one-way ANOVA, with Duncan’s multiple comparisons test. A probability value of < 0.05 was considered significant. (*) indicates the significance between control and treatment groups. *P < 0.05; **P < 0.01. (#) indicates significance between the AMP- and Van-treated groups, with same drug dose. ^#^*P* < 0.05; ^##^*P* < 0.01.

To further elucidate its *in vivo* activity, the efficacy of H2 was determined in a mouse mastitis model. The results showed that after treatment with H2 and NZ2114, bacterial numbers in the mammary glands decreased significantly by 3.96- and 3.58-log, respectively (Fig. 6). A 1.59-log reduction in bacterial cell number was observed in the vancomycin-treated group (Fig. 6). These data indicated that the efficacy of H2 against *S. aureus* in mice is equal to that of NZ2114, but superior to that of vancomycin.

**Figure 7:**
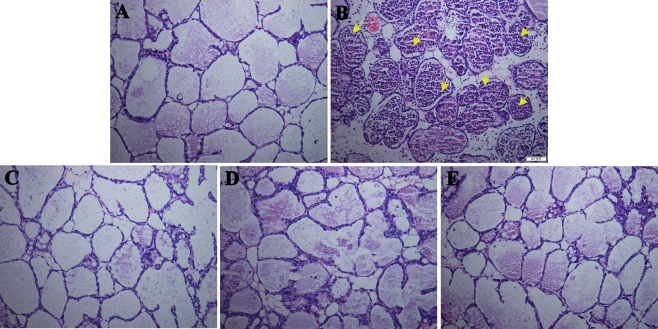
Effects of H2 and NZ2114 on mammary tissues (HE, × 100). The fourth pair of mouse mammary glands was injected with *S. aureus* E48 (1 × 10^5^ CFU per mammary gland) and then treated with 100 μg of the peptides or vancomycin at 4 h after the challenge. The mammary glands tissues were collected from mice at 24 h, fixed in formalin buffer, stained with HE and observed under a microscope. (**A**) Control. (**B**) *S. aureus* + PBS treatment. (**C**) *S. aureus* + H2 treatment. (**D**) *S. aureus* + NZ2114 treatment. (**E**) *S. aureus* + vancomycin treatment. It appeared to no histopathological changes in the blank control group (**A**), the infiltration of immune cells in the negative control (**B**) and alleviation of pathological changes in the treatment control (**C**–**E**). Yellow arrows indicate polymorphonuclear neutrophils.

The mammary tissues of mice inoculated with *S. aureus* were stained with hematoxylin and eosin (HE) and observed under a microscope. As shown in Fig. 7A–E, in the blank control group, no histopathological changes were observed in the mammary tissues, with the exception of a swollen luminal area (Fig. 7A). In the bacteria-infected groups, *S. aureus* E48 induced the infiltration of a number of immune cells, including polymorphonuclear neutrophils, in the alveoli (Fig. 7B). After the treatment with the peptides (100 μg/mammary gland) for 24 h, the pathological changes were significantly alleviated by H2, NZ2114, and vancomycin (Fig. 7C–E).

## Discussion

Bovine mastitis is a serious problem that heavily affects dairy industries worldwide^29^. Due to the intracellular infection of *S. aureus*, it is very difficult to completely eradicate this pathogen by using traditional antibiotics, thus leading to a high infection recurrence^1^. In our earlier research, we found that the plectasin derivatives NZ2114 and MP1102 had a potent antibacterial activity against intracellular *S. aureus*; thus, they may be promising antibiotic alternatives^15,22^. In this study, the intracellular activity of the NZ2114 derivative H2 against different *S. aureus* strains (MRSA ATCC43300, and the clinical bovine mastitis isolates *S. aureus* CVCC3051 and E48) was assessed in bovine mammary epithelial MAC-T cells and a mastitis mouse model.

The *S. aureus* strains can induce the formation of cup-like and pseudopod-like structures in bovine mammary epithelial cells, which extend around the bacteria and engulf the pathogens^7,30,31^. In this study, most intracellular *S. aureus* cells were found in membrane-bound vacuoles in MAC-T cells (Fig. 1); thus, they may escape from being killed by antibacterial drugs. Moreover, the three *S. aureus* strains could survive and proliferate in MAC-T cells (Fig. 1), which was consistent with findings from previous studies^7,31,32^.

It has been demonstrated that polyhexamethylene biguanide, plectasin, NZ2114, and MP1102 have a high efficiency against intracellular *S. aureus* in keratinocytes, human THP-1 and mouse RAW264.7 macrophages^17,21,22,33^. The bovine mammary epithelial MAC-T cells have been widely used to study the physiological function of breast tissues, screen for effective drugs, and simulate the actual conditions of bovine mastitis^23^. The results of this study showed that H2 displayed a strong ability to kill the three different internalized *S. aureus* strains in MAC-T cells, which was equal to that of NZ2114, but superior to that of vancomycin (Fig. 5). Vancomycin, which has the same mechanism of action as plectasin, was used as the positive control in our study, because it is one of the most extensively used antibiotics and the last therapeutic option for treating *S. aureus* infection^34^. Noticeably, the efficiency of H2 and NZ2114 in MAC-T cells (94–99% reduction of bacterial load) is far superior to that of NZ2114 in RAW264.7 cells (63–99% reduction)^22^. This may be attributed to the higher penetration efficiency of these peptides in MAC-T cells (90%) (Fig. 4) than in macrophages (10%)^22^, which may be related to the different internalization rates of the peptides in different cells^25,34^.

After entering the MAC-T cells, the FITC-labeled peptides were distributed in cells in a punctate manner (Fig. 3), suggesting that the peptides are taken up by endocytosis. Moreover, we also found that H2 and NZ2114 could enter the MAC-T cells in a dose-dependent manner and were accumulated in the cytoplasm (Fig. 3), which was similar to the case for polyhexamethylene biguanide, hLF, R9, and Tat11 peptides^25,33,35^. The entrance of the intracellular peptides into cells was enhanced extensively when the peptide concentrations were increased from 2.5 to 25 μg/ml; this was consistent with the results of the flow cytometry analysis (Fig. 4A,B). Analysis of the preliminary mechanisms of drug internalization by using different endocytosis inhibitors showed that the internalization of H2 and NZ2114 was mainly blocked by low temperature and chlorpromazine (Fig. 4C,D). MβCD and nocodazole did not impact the uptake of peptides. Accordingly, it was deduced that H2 and NZ2114 can enter MAC-T cells mainly in an energy- and temperature-dependent manner^25^. In this work, clathrin-mediated endocytosis is the major route of internalization of peptides in MAC-T cells, which is different from the case for certain cell-penetrating peptides (such as Tat11, α1H and α2H)^35,36^ and NZ2114, which were internalized in RAW264.7 cells by both clathrin-mediated endocytosis and micropinocytosis, and from the entry of polyhexamethylene biguanide into cells, which occurs via dynamin-dependent endocytic pathways^37^. This indicated that the same peptide may have various mechanisms and efficiencies of internalization in different cells, thus leading to a diversity of the intracellular antibacterial ability. Additionally, the entrance of H2 and NZ2114 into MAC-T cells was not prevented by heparin, chondroitin sulfate, and dextran sulfate which suggests that the internalization of H2 and NZ2114 does not require the soluble glycosaminoglycans, which is inconsistent with the findings of a previous study, which stated that the uptake of the human immunodeficiency virus type 1 (HIV-1) trans-activator of transcription (Tat11) peptide (residues 48–57) requires cell-surface heparan sulfate proteoglycans^36,38^. Other internalization mechanisms need to be examined in further studies.

It has been demonstrated that *S. aureus* has a high adherence to bovine mammary epithelial cells when cultured in milk serum to mimic the *in vivo* conditions more closely, which may protect pathogen from being phagocytized^39^. Meanwhile, the virulence towards mice of *S. aureus* was enhanced upon growth in milk whey due to the increased clotting activity in bovine plasma and the production of protease^40^. In this study, the *S. aureus*-induced mastitis mouse model was successfully used to test the *in vivo* activity of H2, although there are some differences between mouse and bovine mammary glands. A few studies have demonstrated that the interaction of *S. aureus* with the host cells in mouse mammary glands is similar to that in case of dairy cows^6^. Additionally, in order to investigate the efficiency of H2, in this study, the mouse mastitis model was treated with a lower dose (100 μg per mammary gland) of H2 than that in previous studies (100–1200 μg per mammary gland). The results showed that similar to the findings for the *in vitro* intracellular bactericidal activity (Supplementary Table S3 and Fig. 5c), H2 could kill multidrug-resistant *S. aureus* E48 cells more effectively than vancomycin in the mouse mammary glands (Fig. 6). The lower efficiency of vancomycin *in vivo* may be associated with the presence of body fluids such as milk and serum, which may reduce the antibacterial activity of drugs^23^. Additionally, after treatment with the peptides or vancomycin, polymorphonuclear neutrophils disappeared from the alveoli (Fig. 7), which is consistent with the findings from previous studies^23^. It was also observed that the peptides NZ2114/H2 and vancomycin can protect the mammary glands of the mice from *S. aureus* infection. Meanwhile, no obvious damage to the bacterial cells was found following treatment with the peptides and vancomycin in mammary cells and tissues, indirectly revealing that NZ2114 and H2 have no toxicity at the concentration of 100 μg/gland. This indicated that the plectasin-derived peptide H2 is more promising for use as a topical injection than in transitional therapy for bovine mastitis. Additionally, in some previous studies, it has been demonstrated that the synthetic peptide Bac2A (RLARIVVIRVAR), which is derived from bovine bactenecin, displays potent antibacterial activity against Gram-positive bacteria (MICs of 0.25–16 μg/ml); meanwhile, it has minor effects on host immune responses^41–44^, making it appealing as a therapeutic alternative to antibiotics for mastitis treatment. Another promising candidate used for mastitis treatment is the Bac2A derivative IDR-1018 (VRLIVAVRIWRR); it showed potent activity against *S. aureus* (MIC, 5 μg/ml) and significantly reduced the LPS-induced pro-inflammatory response, indicating that it is one of the strongest synthetic immunomodulatory peptides. These characteristics make IDR1018 an ideal candidate for the treatment of mastitis^44,45^. However, there are some limitations associated with its use, such as expensive production costs, *in vivo* instability, and some cytotoxicity at high concentrations; further studies are required to fully understand and evaluate the biological significance of these peptides^45^.

In summary, H2 displayed a lower cytotoxicity towards bovine mammary epithelial cells than its parental peptide NZ2114. The antibacterial activity of H2 was not reduced by cathepsin B. It entered the bovine mammary epithelial MAC-T cells mainly through clathrin-mediated endocytosis in a dose-dependent manner. H2 showed intracellular bactericidal activities against MRSA and the clinical *S. aureus* isolates in mammary epithelial cells and a mouse mastitis model; its action was found to be superior to that of vancomycin. These findings suggested that H2 is a safe and efficient intracellular antibacterial drug, and may be one of the potential candidates for intramammary therapy in cases of intracellular *S. aureus*-induced clinical mastitis.

## Materials and Methods

### Bacterial strains, cell lines, and mice

The MRSA ATCC43300 strain was purchased from the American Tissue Culture Collection (ATCC). The *S. aureus* CVCC3051 (*spa* type t3297) strain, which was isolated from bovine clinical mastitis milk samples by the Northeast Agricultural University, was purchased from China Veterinary Culture Collection (CVCC) (Beijing, China); it is referred to as a biosafety III strain (Supplementary Table S2). The other clinical mastitis isolate *S. aureus* E48^23^ and MAC-T cells were provided by Professor Zhao, College of Animal Science and Technology, Northwest A&F University (Yangling, China). All strains were cultured in Mueller–Hinton (MH) medium at 37 °C. The MAC-T cells were cultured in DMEM (containing 10% FBS (Invitrogen Trading (Shanghai) Co., Ltd.) and 1% penicillin/streptomycin (Invitrogen Trading (Shanghai) Co., Ltd.). All antibiotics (including virginiamycin, aureomycin, bacitracin zinc, lincomycin, and vancomycin) were obtained from the China Institute of Veterinary Drug Control and Dalian Meilun Biotech and Sangon Biotech (Shanghai) Co., Ltd.. The four-day post-parturient specific-pathogen-free (SPF) ICR mice were purchased from Beijing Vital River Laboratory Animal Technology Co., Ltd. (Beijing, China).

### Intracellular MAC-T infections with multidrug-resistant *S. aureus* strains

The antimicrobial susceptibility and SCC*mec* or *spa* typing of the clinical mastitis *S. aureus* isolates CVCC3051 and E48 were performed as described in the Supplementary information section.

The MAC-T cells were cultivated at 37 °C in conditions of 5% carbon dioxide, and infected with MRSA ATCC43300, and *S. aureus* CVCC3051 and E48 as described previously, with some modifications^46,47^. First, cells (2.5 × 10^5^ cells/ml) were seeded into a 12-well plate (750 μl/well) and cultured in DMEM with 10% FBS (without antibiotics) for 24 h. Then, 750 μl of a suspension of mid-log phase *S. aureus* cells (2.5 × 10^7^ CFU/ml) was added into each well. After incubation for 0.5 h, the cells were washed twice with PBS and incubated with 50 μg/ml lysostaphin for 1 h to remove the extracellular bacteria. The cells were then fixed and visualized as described previously^48^.

### Effect of cathepsin B on the extracellular activities of H2

The effect of the phagolysosomal enzyme cathepsin B on the antibacterial activities of H2 against *S. aureus* was evaluated according to a previous method^46^. In brief, 320 μg/ml H2 or NZ2114 was incubated with a 16 μg/ml enzyme solution (20 mM sodium acetate, 1 mM EDTA, and 5 mM, L-cysteine, pH 5.0) for 1 h at 37 °C, and their MICs against *S. aureus* were determined by the broth microdilution method.

### Cytotoxicity of H2 towards MAC-T cells

The cytotoxicity of H2 and NZ2114 towards MAC-T cells was tested by a colorimetric method, i.e. the MTT assay, as described previously^22^.

### Translocation, subcellular distribution, quantification, and preliminary mechanism of H2 uptake into MAC-T cells

MAC-T cells were seeded into 12-well plates (1.875 × 10^5^ cells/well) and cultured for 24 h at 37 °C. The H2 or NZ2114 solutions were added into the cell suspensions with the final concentration of 2.5, 25, 250 μg/ml, respectively, followed by incubation for an additional 24 h. After washing and staining with 50 μg/ml PI for 10 min, the membrane integrity of cells was analyzed using a FACS Calibur Flow Cytometer (BD, USA)^48^.

MAC-T cells were grown on a confocal dish. The FITC-labeled H2 and NZ2114 peptides (2.5, 25, and 50 μg/ml) were added into the cell cultures, followed by incubation for 24 h. After washing with PBS, the cells were then stained with Hoechst 33342 (5 μg/ml, Invitrogen, nuclear stain) and wheat germ agglutinin (WGA)-conjugated Alexa Fluor 555 (5 μg/ml, Invitrogen, membrane stain) for 10 min and observed using confocal microscopy^37^.

To quantify the uptake of the peptides, the cells were seeded and incubated with the FITC-labeled peptides (0.25, 2.5, and 25 μg/ml), as described above. To quench the membrane-bound FITC-labeled peptides, the cells were treated with 0.04% trypan blue (Invitrogen, UK). The fluorescence intensity of 10,000 gated cells was then analyzed by flow cytometry^33,37^.

To explore the preliminary mechanism of H2 uptake into cells, the MAC-T cells were preincubated with different endocytosis inhibitors (6 μg/ml chlorpromazine, 5 mM MβCD, 3 mM amiloride, and 20 µM nocodazole) at 37 °C for 1 h. FITC-labeled H2 or NZ2114 (25 μg/ml) were then added, followed by incubation for another 6 h. The cells treated with the FITC-labeled peptides at 4 °C and 37 °C were used as the negative and positive controls, respectively. The membrane-bound FITC-labeled peptides were quenched, and the cell fluorescence intensity was measured as described above^35^.

MAC-T cells were incubated with 25 μg/ml of the FITC-labeled peptides and 10 μg/ml of soluble glycosaminoglycans (heparin, chondroitin sulfate, and dextran sulfate) for 6 h, followed by the measurement of the fluorescence intensity, as described above^38^.

### *In vitro* efficacy of H2 in MAC-T cells

To determine the intracellular activity of H2, MAC-T cells were infected with *S. aureus*, as described above. The survival of intracellular *S. aureus* was evaluated 24 h after incubation with H2, NZ2114, or vancomycin (0.25, 1.25, 2.5, 12.5, and 25 μg/ml). After washing with PBS, MAC-T cells were lysed with Hanks’ buffered saline solution (0.1% bovine serum albumin and 0.1% Triton-X)^23,46^. The intracellular bacteria were diluted by PBS and plated for CFU determination at 0 h and 24 h.

### *In vivo* efficacy of H2 in the mouse mastitis model

The SPF ICR mice were bred in the appropriate conventional animal care facilities and all experiments were performed in accordance with the Animal Care and Use Committee of the Feed Research Institute of the Chinese Academy of Agricultural Sciences (CAAS). The protocols were approved by the Laboratory Animal Ethical Committee and the Inspection of the Feed Research Institute of the CAAS (AEC-CAAS-20090609).

The efficacy of H2 against *S. aureus* was evaluated in a mouse mastitis model, as described previously^23,49^. To deplete the milk in mammary glands, the pups were nursed for 1 h. The lactating mice were anesthetized by the intraperitoneal injection of tribromoethyl alcohol. The fourth pair of mammary glands of each mouse was subjected to intramammary challenge with *S. aureus* E48 (1 × 10^5^ CFU in 100 μl PBS per mammary gland). In total, 20 mice were randomly divided into the PBS-, H2-, NZ2114-, and vancomycin-treated groups. The glands were injected with 100 μg of the peptides or vancomycin (dissolved in 100 μl PBS) at 4 h after the challenge. Twenty-four hours later, the mice were euthanized, and their mammary glands were dissected, weighed, and homogenized; the bacterial cell numbers were counted as described above. The mammary gland tissues of mice from each group were fixed in formalin buffer and their histopathological changes were then examined under a light microscope.

### Statistical analyses

All statistical analyses were performed using IBM SPSS Statistics 21.0 and one-way repeated analysis of variance (ANOVA) with Duncan’s test. The data were expressed as the means ± SDs. The differences were considered to be significant at *P* < 0.05.

## Supplementary information

Supplementary information
