# Supplementary material for: Internalization, distribution, and activity of peptide H2 against the intracellular multidrug-resistant bovine mastitis-causing bacterium Staphylococcus aureus
Source: Sci Rep. 2019 May 28;9:7968. doi: 10.1038/s41598-019-44459-x (PMC6538662; doi:10.1038/s41598-019-44459-x)
Supplement: Supplementary file 1 — Supplementary information [file 41598_2019_44459_MOESM1_ESM.pdf]

**Internalization, distribution, and activity of peptide H2 against the intracellular multidrug-resistant bovine mastitis-causing bacterium *Staphylococcus aureus***

**Xiao Wang [1,2*], Da Teng [1,2*], Xiumin Wang [1,2], Ya Hao [1,2], Huixian Chen [1,2], Ruoyu Mao [1,2**] & Jianhua Wang [1,2**]**

**[1] Key Laboratory of Feed Biotechnology, Ministry of Agriculture, Beijing 100081, People's Republic of China. [2] Gene Engineering Laboratory, Feed Research Institute, Chinese Academy of Agricultural Sciences, Beijing 100081, People's Republic of China. * These authors contributed equally. ** Correspondence and requests for materials should be addressed to R.Y.M. (maoruoyu@caas.cn) and J.H.W. (email：2681298635@qq.com)**

## SUPPORTING INFORMATION

### Supplementary 1: Materials and Methods

**Antimicrobial susceptibility testing of *S. aureus*.** Antimicrobial susceptibility testing of *S. aureus* (subclinical isolate *S. aureus* CVCC3051 and E48) was performed by Kirby Bauer disc diffusion method according to the standards procedures recommended by Clinical Laboratory Standards Institute (CLSI 2012)[1,2]. The antibiotics used in this study were same with our previous research[3].

**SCC*mec* and *spa* typing of *S. aureus*.** The bacteria DNA was extracted and then used as a template in a 50-μl PCR. The SCC*mec* and x region of the *spa* gene was amplified by PCR with the same primers as Wang[3]. The x region of the spa genes was sequenced by Sangon Biotech (Shanghai, China) and the types were determined with the database accessible via http://spa.ridom.de/spatypes.shtml.

**FITC-labeled peptides.** The FITC-labeled peptides were carried out in ChinaPeptides Co., Ltd. (Shanghai, China) as previously described[4].

### References

[1] Kahsay, A., Mihret, A., Abebe, T. & Andualem, T. Isolation and antimicrobial susceptibility pattern of *Staphylococcus aureus* in patients with surgical site infection at Debre Markos Referral Hospital, Amhara Region, Ethiopia. *Arch Public Health* **72**, 16 (2014).

[2] Wikler, M. A. Performance standards for antimicrobial sensitivity testing: seventeenth informational supplement. *CLSI* **26**, 1–177 (2007).

[3] Wang, X., *et al.* Increased intracellular activity of MP1102 and NZ2114 against *Staphylococcus aureus in vitro* and *in vivo*. *Sci Rep* **8**, 4204 (2018).

[4] Sun, X. X., *et al.* Fluorescence characterization of the thermal stability of collagen mimic peptides. *Chin J Chem* **28**, 963–967 (2017).

**Supplementary 2: Figure**

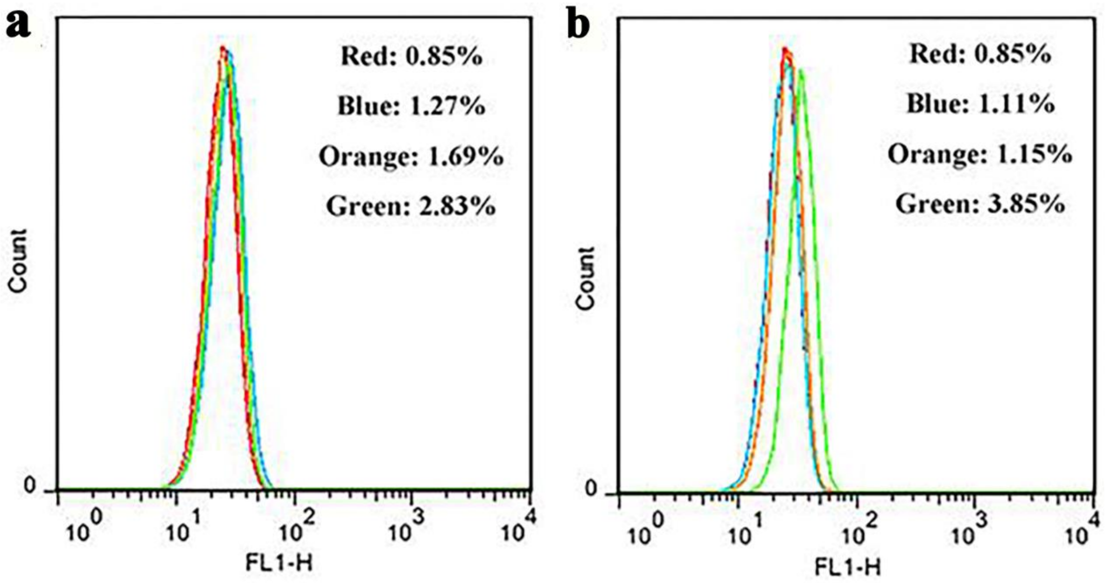


**Fig. S1**

**Figure 1** Effect of H2 and NZ2114 on the cell membrane of MAC-T cells. Cells were incubated with different concentrations of H2 **(a)** and NZ2114 **(b)** for 24 h prior to staining with 5 μg/ml PI at 37 ℃ for 10 min. PI fluorescence in MAC-T cells was measured by flow cytometry. Red line: control; blue line: 2.5 μg/ml peptides; orange line: 25 μg/ml peptides; green line: 250 μg/ml peptides.

**Supplementary 3: Tables**

**Table 1** Antimicrobial susceptibility patterns of *S. aureus* CVCC3051 and E48

| Antibiotics | *S. aureus* CVCC3051 | *S. aureus* E48 |
|---|---|---|
| Vancomycin | S | S |
| Lincomycin | I | I |
| (AMX/20) Amoxicillin | S | S |
| Ciprofloxacin | S | I |
| Amikacin | S | S |
| Ampicillin | S | R |
| Oxacillin | S | S |
| Erythrocin | I | I |
| Tetracycline | S | S |
| Bacitracin | R | R |
| Norfloxacin | S | S |
| Sulfisoxazole | R | R |
| Neomycin | I | I |
| Azithromycin | S | S |
| Kanamycin | I | I |
| Streptomycin | R | R |
| Cefotaxime | S | I |
| Gentamicin | S | S |
| Chloramphenicol | S | S |
| Cefazolin | S | S |
| Penicillin | S | S |

R: resistant; I: Intermediate; S: susceptible.

**Table 2** Sources, types and susceptibilities of *S. aureus* CVCC3051 and E48

| ***S. aureus*** | **Source** | **SCC*mec* type** | ***Spa* type** | **Antibiotic resistance profile** | **Biosafety level** |
|---|---|---|---|---|---|
| *S. aureus* CVCC3051 | Clinical isolate | - | t3297 | Bacitracin, sulfisoxazole streptomycin | III |
| *S. aureus* E48 | Clinical isolate | - | t3583 | Ampicillin, bacitracin sulfisoxazole, streptomycin | N |

-: negative. N: unknown

**Table 3** Effect of cathepsin B on the MICs of H2, NZ2114 and vancomycin toward three different *S. aureus* strains

| **Drugs** | **MICs (μg/ml) AMP** | | | **MICs (μg/ml) AMP + cathepsin** | | |
|---|---|---|---|---|---|---|
| | **ATCC43300** | **CVCC3051** | **E48** | **ATCC43300** | **CVCC3051** | **E48** |
| H2 | 0.5 | 0.5 | 1 | 0.5 | 0.5 | 1 |
| NZ2114 | 0.25 | 0.5 | 1 | 0.25 | 0.5 | 1 |
| Vancomycin | 1 | 1 | 1 | 1 | 1 | 1 |
